# Social isolation: main dermatosis and the impact of stress during the COVID-19 pandemic

**DOI:** 10.31744/einstein_journal/2022AO6320

**Published:** 2022-03-17

**Authors:** Carolina Soutto Mayor Mangini, Rossana Cantanhede Farias de Vasconcelos, Eduarda Villela Rosa Rodriguez, Isabela Romeu Lorenzon de Oliveira

**Affiliations:** 1 Universidade Santo Amaro São Paulo SP Brazil Universidade Santo Amaro, São Paulo, SP, Brazil.

**Keywords:** Skin diseases, Stress, psychological, Coronavirus infections, COVID-19, Pandemics, Social isolation

## Abstract

**Objective:**

To analyze the pattern of triggering and exacerbation of dermatological diseases between March and July 2020 and to compare this pattern to the corresponding period of 2019.

**Methods:**

This was a quantitative, descriptive, comparative and documentary study that was carried out through the retrospective analysis of medical records (March to July 2019 and 2020) of individuals assisted at a private dermatology practice service located in the southern area of the city of São Paulo (SP).

**Results:**

We evaluated 992 medical consultations in 2019 and 1,176 in 2020. In 2020, we observed a significant increase in cases of telogen effluvium (276%), psoriasis (1,400%), atopic dermatitis (178%), seborrheic dermatitis (200%), herpes zoster (1,200%) and vitiligo (433%). All diseases had stress as a possible initial trigger. In addition, fragile nail syndrome and contact dermatitis, pathologies associated with behavioral measures, also had an important increase in the prevalence (6,400% and 5,500%, respectively). However, the number of aesthetic procedures decreased by approximately 54% during the pandemic period.

**Conclusion:**

During the pandemic period, the pattern of incidence of dermatoses had changed compared with the previous year. An emphasis was observed on diseases triggered by a psychological component, as well as those pathologies that have behavioral measures as the main cause. For this reason, the impacts of COVID-19 is greater than only among those infected.

## INTRODUCTION

The World Health Organization (WHO) declared, on January 30, 2020, the outbreak caused by the new coronavirus as a public health emergency of international concern.^(1)^ This injury is caused by the severe acute respiratory syndrome coronavirus 2 (SARS-CoV-2), a betacoronavirus that affects the lower portion of the respiratory tract and has widely variable symptomatology, which can be asymptomatic, oligosymptomatic or even lethal.^(1)^

Emergency measures, such as the closure of schools, universities and non-essential establishments, as well as the suggestion of quarantine and social isolation, have been proposed by the Brazilian Ministry of Health.^(2)^ Elderlies, individuals with chronic, pulmonary and/or cardiovascular diseases are more likely to develop the severe forms of the disease. However, to prevent the spread of the disease, it is paramount that the entire population to remain in social isolation, since a large part of the infections occur through asymptomatic individuals.^(2)^

A public health emergency, associated with social isolation, may trigger pathological stress, generalized anxiety disorders, and depression.^(3,4)^ In this scenario, individuals are more vulnerable to the establishment or exacerbation of dermatoses, which may result in a worsening of quality of life.^(5)^

Emotions and pathologies are directly related, by means of connections, between the neurological, neuroendocrine and immunological systems.^(5,6)^ Dermatoses triggered by events that involve the interaction between skin and mental changes are called psychodermatoses and they are influenced by factors such as stress, fear, negative thoughts and anxiety.^(6,7)^ Some cutaneous manifestations included in this subgroup are: telogen effluvium, alopecia areata, psoriasis, atopic dermatitis, seborrheic dermatitis, and vitiligo.^(7-9)^

As a form of viral containment, certain behavioral measures, such as constant use of face masks and alcohol gel, were established.^(8)^ Although essential for the control of the worsening, these practices resulted in the appearance of certain dermatoses, such as brittle nail syndrome, contact dermatitis, and burns.^(9,10)^ Finally, certain dermatological lesions have been described as possible manifestations of SARS-CoV-2, which highlights the importance of the differential diagnosis of skin diseases in the context of the pandemic.^(11)^

## OBJECTIVE

To analyze the pattern of triggering and exacerbation of dermatoses in the social isolation period recommended by the Brazilian Ministry of Health in the COVID-19 pandemic, and to compare this pattern to the diagnoses established during the corresponding months of the year of 2019.

## METHODS

This was a quantitative, descriptive, comparative and documental research carried out through the retrospective analysis of medical records of individuals assisted at a private dermatology office located in the southern zone of the city of São Paulo (SP) from March to July 2019 and 2020. This study was approved by the Ethics Committee of the *Universidade Santo Amaro* (UNISA) # 4.237.898 and CAAE: 36817220.8.0000.0081. Due to the amount of medical records analyzed and the restrictions necessary for the prevention of the new coronavirus, it was not possible to have the patients’ consent. The reason for non-submission of informed consent for was presented to and approved by the Ethics and Research Committee.

The prevalence of psychodermatoses was measured during the pandemic period and it was compared to the corresponding period of the year 2019. Medical records of patients at any age range and sex, with or without associated pathology, were included. Data collection was performed from March to August 2020 and, once in contact with the current dermatoses, their prevalence in the year 2019 was analyzed. Considering that the data referring to 2019 had only comparative value, not all dermatological consultations of that same year were accounted for the statistics. We included only the dermatoses observed in 2020. Categorical data (proportion of consultations of each disease in 2019 and 2020) were described by frequency and percentage. To assess whether there was a difference in the proportion of consultations in 2019 and 2020 for each disease, the test of proportions or equity was used.

## RESULTS

**Figure 1:**
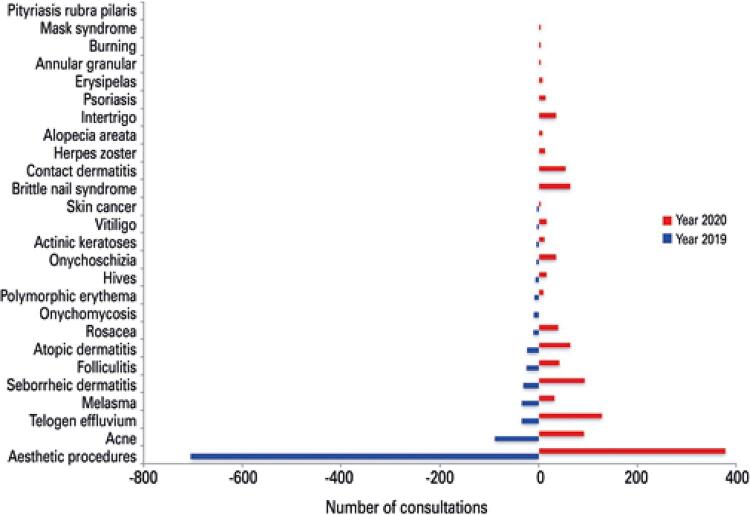
Comparison of dermatological consultations between 2019 and 2020

A total of 992 consultations in 2019 and 1,176 in 2020 were evaluated. When the proportion of consultations were compared between 2019 and 2020 (Table 1 and Figure 1), a higher proportion of consultations in onychomycosis and aesthetic care was seen in 2019. In 2020, there was a significant increase in cases of telogen effluvium (276%), atopic dermatitis (178%), seborrheic dermatitis (200%), shingles (1,200%), and vitiligo (433%) – all conditions related to stress as a possible initial triggering. Furthermore, brittle nail syndrome and contact dermatitis, pathologies triggered by behavioral measures also had an important increase in their prevalence (6,400% and 5,000%, respectively). On the other hand, the number of cosmetic procedures decreased by approximately 54% during the pandemic period.

**Table 1:** Comparison between number of consultation in 2019 and 2020 for each disease

Diagnosis	2019	2020	Total	p value

	n (%)	n (%)	n (%)	
Telogen effluvium	34 (20.98)	128 (79.01)	162 (100)	<0.001*
Seborrheic dermatitis	31 (25)	93 (75)	124 (100)	<0.001*
Acne	89 (49.17)	92 (50.82)	181 (100)	0.833
Fragile nail syndrome	1 (1.53)	64 (98.46)	65 (100)	<0.001*
Contact dermatitis	1 (1.78)	55 (98.21)	56 (100)	<0.001*
Folliculitis	24 (36.36)	42 (63.63)	66 (100)	0.003*
Atopic dermatitis	23 (26.43)	64 (73.56)	87 (100)	<0.001*
Rosacea	11 (21.56)	40 (78.43)	51 (100)	<0.001*
Onycholysis	4 (10.25)	35 (89.74)	39 (100)	<0.001*
Melasma	34 (51.51)	32 (48.48)	66 (100)	0.861
Urticaria	6 (27.27)	16 (72.72)	22 (100)	0.006*
Vitiligo	3 (15.78)	16 (84.21)	19 (100)	<0.001*
Shingles	1 (7.14)	13 (92.85)	14 (100)	<0.001*
Actinic keratosis	4 (25)	12 (75)	16 (100)	0.013*
Erythema polymorphous	8 (44.44)	10 (55.55)	18 (100)	0.738
Alopecia areata	1 (11.11)	8 (88.88)	9 (100)	0.004*
Skin cancer	1 (16.66)	5 (83.33)	6 (100)	0.083
Onychomycosis	9 (81.81)	2 (18.18)	11 (100)	0.010*
Aesthetic treatments	705 (65.09)	378 (34.9)	1,083 (100)	<0.001*
Total of consultations	992 (45.75)	1,176 (54.24)	2,168 (100)	<0.001*

* p<0.05.

In 2020 a higher proportion of consultations were observed for telogen effluvium, seborrheic dermatitis, brittle nail syndrome, contact dermatitis, folliculitis, atopic dermatitis, rosacea, onycholysis, urticaria, vitiligo, shingles, actinic keratosis, and alopecia areata.

## DISCUSSION

The pandemic caused by coronavirus disease 2019 (COVID-19) has not only affected individuals who have been infected. Many of those who have not been infected are also being indirectly impacted by the global scenario, by the widespread exposure to stressful variables, such as the social isolation recommended by the WHO.^(10)^

The drastic change that occurred in the routine of Brazilians with the situation imposed by the pandemic such as the closing of non-essential establishments, and the massive amount of news broadcast daily by the media about the world scenario are examples of factors that can trigger or amplify a clinical picture of stress among individuals.^(11)^

A survey that was carried out with 45,161 Brazilians during the pandemic concluded that 40.4% of the interviewees were often or always sad or depressed, 52.6% were always or almost always anxious or nervous, 43.5% reported onset of sleep problems, and 48.0% felt worsening of pre-existing sleep problems.^(3)^ Furthermore, the study by Brooks et al.^(4)^ revealed a considerable percentage increase in the impairment of mental health of individuals who went through quarantine in relation to those who did not go through this experience.

The first scientist who defined stress, paying attention to its biological dimension, was Hans Selye, a Hungarian endocrinologist.^(12)^According to him, stress causes changes in the structural and chemical composition of the body, that is, it is inherent to every disease.^(12)^ Since then, the importance of this feeling on physiological processes has been widely studied.

Psychological stress activates the hypothalamic-pituitary-adrenal axis and the sympathetic nervous system.^(5)^ In addition, this induces the secretion of different neurotransmitters, cytokines and hormones, which reach cutaneous receptors and can trigger or worsen several dermatoses. Stress-related issues affect innate immunity, adaptive immunity, and skin barrier homeostasis.^(5)^

Acute and chronic stress can occur in different ways in individuals.^(5)^ In a specific situation, the protective and destructive aspects of the stress response must be precisely understood, since this data is crucial to evaluate the relevance of stress in the onset or exacerbation of skin diseases and, subsequently, to integrate this understanding for a better diagnosis, procedure and treatment.^(5)^

According to the survey conducted, hair loss was reported by 136 patients in 2020. Of these, 128 were diagnosed with telogen effluvium, a pathology in which, in response to situations of physical and emotional stress, the hair follicle is reprogrammed to cease hair growth prematurely.^(13)^Eight patients presented alopecia areata, a disease that, despite its multifactorial etiology, is aggravated in the presence of atopy and psychic trauma.^(14)^

The onset or exacerbation of acne showed significant prevalence, but it was similar to that observed in 2019, which can be explained by the multifactorial nature of the disease. Catecholamines are the main neurotransmitters released in stress situations, and they are able to activate substances by the sebocytes, making the environment hyperseborrheic and anaerobic, therefore, favoring the proliferation of *Cutibacterium* acnes.^(15)^Furthermore, neurotransmitters influence the formation of biofilm by these bacteria, which increases the inflammatory potential of the strains and increases their pathogenicity.^(15)^Patients with severe acne have lower serum levels of brain-derived neurotrophic factor (BDNF), a biomarker related to the development of chronic stress, anxiety, and depression.^(15)^

Erythemato-descaling lesions, whose exacerbations are associated with stress,^(16-18)^were also observed. In 2020, 93 patients had seborrheic dermatitis, 14 had psoriasis, and two had pityriasis rubra pilaris.

High scores on the Dermatology Life Quality Index (DLQI), referring to quality of life in dermatology, and the Psoriasis Area Severity Index (PASI), referring to the psoriasis area severity index, are generally related to depression, psychological stress, and anxiety.^(16)^

Eczematous lesions, such as atopic dermatitis, showed an increase of 41% points compared with 2019. Activation of the aryl hydrocarbon receptor (AHR), a dioxin receptor, also occurs as a function of oxidative stress, and even when it is caused by psychological stress.^(16)^This receptor is associated with the onset or exacerbation of pathologies, since it binds to environmental polychromatic hydrocarbons and dioxins with high affinity. These factors are in force in patients with psoriasis and contact dermatitis due to the their signaling pathways.^(16)^

During the pandemic, vitiligo was observed in 16 patients. Stress increases the secretion of neuroendocrine hormones and autonomic neurotransmitters, causing the immune system to be modified and the neuropeptide-rich brain regions to be altered, therefore, have their conditions changed.^(19)^

Chronic stress may also increase skin cancer susceptibility by shifting the balance of protective versus suppressive immune responses.^(20)^ There is suppression of type 1 cytokines, interleukin (IL)-12p40 gene expression, CCL27/CTACK, CD3ε, and infiltration of TCD4+ and CD8+ cells at the sites of emergence, which may affect tumor progression.^(20)^

Rosacea, observed in 40 individuals, has trigger such as hormonal changes, medications, emotional stress, and psychological factors.^(21)^ As well as shingles,^(22)^ which showed an increase of 85.71% in 2020 compared with 2019.

Behavioral changes related to social isolation were also responsible for a large portion of the recorded complaints. In addition to stress and anxiety, decubitus for prolonged periods, sedentarism, dietary changes, progression of obesity, decompensation of diabetes, and increased blood pressure were some of the behavioral and metabolic changes experienced during this period.^(23)^ These comorbidities may result in relative immunosuppression of the organisms, added to venous insufficiency and other cardiovascular disorders,^(23)^being possible risk factors for several dermatoses, such as erysipelas, intertrigo and folliculitis. This disease is observed 1.75 times more in patients analyzed in 2020, which has its manifestation enhanced in the presence of trauma, perspiration, friction and occlusion.

Medications used in the treatment of various dermatoses can also cause erythema polymorphus, a potentially serious condition that requires immediate dermatological intervention,^(24)^ which showed a higher prevalence in 2020.

The increased use of electronic devices by the population in the quarantine period is extremely relevant, since there is consequently greater exposure to blue light, currently much discussed in relation to the risk of oxidative stress and pigmentation.^(25)^

Other behavioral changes described are also associated with prevention of the new coronavirus, such as increased use of alcohol, in its liquid and gel form.^(8)^ The product was recommended to the population, especially when in contact with potentially contaminated people and objects, and in the impossibility of washing hands with soap and water. In March 19, 2020, the *Agência Nacional de Vigilância Sanitária* (ANVISA) made 70% liquid alcohol commercialization more flexible, causing its use to increase exponentially.^(8)^This measure may be associated with the onset of brittle nail syndrome, diagnosed in 5.44% of patients seen in 2020, as well as onychomycosis, found in 2.98%, onychomycosis, and recorded in 0.17%, and even a clinical picture of contact dermatitis, diagnosed in 55 patients. Such diseases had a higher prevalence in the medical records surveyed in 2020 than in the previous year, with the exception of onychomycosis.

In addition to the risks already discussed about 70% liquid alcohol, it is an extremely flammable product, and its improper handling or contact with fire after use can cause burns,^(8)^ a complaint that increased significantly during the period studied.

The incidence of patients affected by onychomycosis was higher in 2019, given that the onset or exacerbation of the pathology is not directly associated with some issues of social isolation. The main risk factors for the establishment of the pathology are tinea pedis, preexisting ungual dystrophy, advanced age, male gender, immunocompromise, peripheral vascular disease, diabetes, and contact with individuals already affected by the dermatosis.^(26)^

In addition to psychosomatic factors and the drop in the immune system, the onset and exacerbation of acne and rosacea have been observed due to the constant use of individual protective face masks, which became mandatory in public and private spaces of public circulation as an attempt to fight the COVID-19 pandemic, and required by law 14.019, of July 2, 2020 to be used.^(27)^ Since then, the emergence of certain dermatoses related to the use of masks has been registered. The skin represents the individual’s primary line of defense against the environment, and this vulnerable to all environmental harmfulness, such as mechanical forces.

For professionals who deal directly with patients and potentially infected environments, the use of Personal Protective Equipment (PPE) is essential.^(7)^ The equipment is responsible for creating a barrier that, in turn, can trigger injuries due to constant friction, such as contact dermatitis and pressure ulcers.^(7)^ This phenomenon was named mask syndrome and refers to the prolonged use of individual protection, subjecting the individual’s skin to vulnerable situations, such as high perspiration and mental stress.^(28,29)^Methods to prevent skin damage have been considered, since, in addition to being painful, they can increase the susceptibility to bacterial, fungal and viral infections, such as coronavirus.^(8)^

Granuloma annulare, urticaria, folliculitis, melasma and polymorphous erythema were aggravations that, despite not having psychological factors or behavioral measures as initial trigger, were recorded during the pandemic period.

Aesthetic-related attendances and procedures also showed significant variation when the years 2019 and 2020 were compared. In 2020, when social isolation was proposed, the demand for aesthetic care reduced drastically, and this fact may be an indicator of a fear experienced by the population during this period. Health professionals recommended that non-essential procedures should be avoided,^(30)^ and therefore most of the cases seen during this period were non-aesthetic complaints.

In addition to skin involvement due to the consequences of social isolation, recent studies have discussed the possible relationship of SARS-CoV-2 infection with dermatological manifestations.^(9)^ Patients infected with the coronavirus presented vesicular, purpuric and morbilliform eruptions; urticaria; erythema perniosum, erythema palmaris and enanthema, and livedoid or necrotic lesions, besides maculopapular eruptions, such as pityriasis roseosa, perifollicular eruption and erythema multiforme.^(9)^

Although some scientific analyses claim that it is too early to consider such findings characteristic and suggestive of COVID-19, identifying a pattern caused by the disease in infected patients is of great value to the medical context during a pandemic. This is especially relevant to help health professionals to recognize oligosymptomatic patients and establish a possible prognosis given that, although the specific test for coronavirus is indispensable for diagnostic confirmation, several regions of Brazil and the world suffer from the scarcity of this diagnostic tool.^(9)^ Therefore, knowledge of these lesions can help in clinical management and epidemiological control. First, physicians should pay attention to the evolution of the patient and to the action to be taken, until the possibility of COVID-19 infection is excluded.^(9)^

## CONCLUSION

During the pandemic of COVID-19, a significant increase in the prevalence of psychodermatoses was observed, among other skin manifestations, when compared to the corresponding previous period in 2019. The telogen effluvium was the most common pathology observed in the period of social isolation, which demonstrated stress as a significant risk factor for the establishment and exacerbation of dermatoses. For this reason, it is possible to conclude that the impacts of the pandemic are greater among infected individuals.

Finally, this study highlights the importance of dermatologists in the prevention, diagnosis and treatment of the diseases addressed and, more than that, in the promotion of the incentive for new studies approaching the correlation of stress and other circumstances established during quarantine due to the establishment of diseases. Therefore, to emphasize the pathophysiology of each one of these disease is crucial for the improvement in medical care delivery for patients.
